# A deep learning approach for fully automated measurements of lower extremity alignment in radiographic images

**DOI:** 10.1038/s41598-023-41380-2

**Published:** 2023-09-06

**Authors:** Ki-Ryum Moon, Byoung-Dai Lee, Mu Sook Lee

**Affiliations:** 1 Division of AI and Computer Engineering, Kyonggi University, Suwon, Republic of Korea; 2 Department of Radiology, Keimyung University Dongsan Hospital, 1035, Dalgubeol-Daero, Sindang-Dong, Daegu, 24601 Republic of Korea

**Keywords:** Information technology, Radiography, Computer science

## Abstract

During clinical evaluation of patients and planning orthopedic treatments, the periodic assessment of lower limb alignment is critical. Currently, physicians use physical tools and radiographs to directly observe limb alignment. However, this process is manual, time consuming, and prone to human error. To this end, a deep-learning (DL)-based system was developed to automatically, rapidly, and accurately detect lower limb alignment by using anteroposterior standing X-ray medical imaging data of lower limbs. For this study, leg radiographs of non-overlapping 770 patients were collected from January 2016 to August 2020. To precisely detect necessary landmarks, a DL model was implemented stepwise. A radiologist compared the final calculated measurements with the observations in terms of the concordance correlation coefficient (CCC), Pearson correlation coefficient (PCC), and intraclass correlation coefficient (ICC). Based on the results and 250 frontal lower limb radiographs obtained from 250 patients, the system measurements for 16 indicators revealed superior reliability (CCC, PCC, and ICC ≤ 0.9; mean absolute error, mean square error, and root mean square error ≥ 0.9) for clinical observations. Furthermore, the average measurement speed was approximately 12 s. In conclusion, the analysis of anteroposterior standing X-ray medical imaging data by the DL-based lower limb alignment diagnostic support system produces measurement results similar to those obtained by radiologists.

## Introduction

Lower limbs, which constitute half of the human body, maintain a stable state of movement by absorbing external forces and shocks while supporting body weight during walking or running. When a deformation persists in one part, considerable orthopedic disabilities, such as differences in the lengths of the two legs or bending and twisting of the lower limbs, may occur^1^. Appropriate alignment of lower limbs is crucial in maintaining correct posture and preventing pain owing to poor posture. However, minor malalignments in the lower limbs can limit body movements and cause muscle tension. When severe malalignment occurs, excessive weight placed on the medial or lateral side of the knee can cause strain on the knee joint, resulting in osteoarthritis. Furthermore, leg length discrepancy may introduce twisted posture while standing or walking and bend the spine, which can cause pain during exercise or prolonged walking. In severe cases, the risk of secondary complications, such as scoliosis, lower back pain, and disk problems, increases significantly. Therefore, the malalignment of lower limbs can severely disrupt day-to-day functions^2^.

Methods to diagnose the presence and degree of lower limb malalignments include measuring the lengths of both legs using a vertical line or posture grid when the patient is standing^3^, obtaining measurements from photographs displaying anatomical landmarks using a goniometer and ruler with lateral lines or square grids^4^, and radiographic examination^5^. However, examinations using goniometers and rulers have several limitations, such as inaccuracy and lack of standardization^6^. Contrastingly, radiographic examination is accurate because malalignments are evaluated based on the joint position and direction of lower limbs using quantitative criteria, including leg-length measurements determined from X-ray radiographs obtained when the patient is standing^6,7^. However, this method is unsuitable for widespread application because it requires specialized knowledge for manipulation and interpretation, which, is time consuming owing to the determination of numerous quantitative parameters^6^. Furthermore, subjectivity is introduced because the observed values of these parameters vary among observers^6,8,9^. Therefore, it is crucial to develop a system that can rapidly and accurately measure the values of parameters determining the alignment of the lower limbs.

Recently, rapid advances in deep learning (DL) technology based on artificial neural networks has expanded its applications, particularly in medical imaging analysis. Using DL technology, excellent results have been achieved in evaluating scoliosis^10^ and assessing lower limb alignments^11–14^. Therefore, this study aimed to evaluate the feasibility of computer-assisted quantification program for measuring the lower extremity alignment. Hence, a DL-based system was developed to rapidly and accurately measure various parameters for automatically determining the alignment of lower limbs, and its diagnostic performance and reliability were evaluated using full-leg radiographs.

## Related research

Methods using DL techniques on radiographs to automatically assess lower limb alignment have been proposed to improve and supplement the manual interpretations of radiologists. To assess leg-length discrepancies in pediatric patients using radiographic images, Zheng et al.^11^ proposed a system using DL techniques. A U-Net with mixed residual blocks was used to segment femurs and tibias in radiographs, followed by leg-length calculation. Furthermore, the measurement of pediatric leg-length on radiographs was automated and performed rapidly using a DL algorithm.

Schock et al.^12^ proposed a DL method for automatically analyzing lower limb alignment by calculating the anatomic–mechanical angle (AMA) and hip–knee–ankle angle (HKAA) using weight-bearing bilateral lower limb radiographs. U-Net^15^, which is generally employed in semantic segmentation techniques, was used to generate the binary mask images required for quantitatively measuring AMAs and HKAAs. Furthermore, various data augmentation techniques were adopted to prevent the overfitting of model.

Tack et al.^13^ proposed a multi-stage approach to localize relevant landmarks for assessing lower limb alignment. First, YOLOv4^16^ was used to detect regions of interest (ROIs) in the entire lower limb radiographs, wherein landmarks within individual ROIs were located using ResNet^17^. Second, the mean radial error was used as a loss function to minimize the regression errors. However, this study is limited in that the performance of ROI extraction by YOLOv4 is inconsistent for radiographs with low contrast, thereby introducing significant errors in the measurement of HKAA.

Finally, Lee et al.^14^ proposed a DL-based system to automatically measure the leg-length using the entire leg radiographs of diverse patients, including those with orthopedic hardware implanted for surgical treatment. The system comprised a four-stage cascade architecture–ROI detection, bone segmentation, landmark detection, and leg-length calculation. For ROI detection and bone segmentation, a customized single-shot multi-box detector^18^ and XY-attention network^19^ were used, respectively. Independent of the orthopedic hardware implanted in the lower extremity limbs of patients, the performance of this system was similar to that of radiologists in terms of accuracy and reliability.

## Material and methods

This retrospective study was approved by the institutional review board of Keimyung University Dongsan Medical Center, where it was conducted (IRB No. DSM-2021-04-063). All methods were performed in accordance with the ethical standards of Helsinki Declaration. Because the data used in this retrospective study were fully de-identified to protect patient confidentiality, the requirement for informed consent was waived by the institutional review board of Keimyung University Dongsan Medical Center.

### Study participants and datasets

The leg radiographs of non-overlapping 770 patients were collected from January 2016 to August 2020 (Innovision; DK Medical Systems Co. Ltd., Seoul, South Korea). Among 770 images, we excluded 320 images featuring at least one artificial joint and images belonging to patients that underwent hip arthroplasty or exhibited skeletal or fibrous dysplasia. The remaining 450 images were used for system development, training, and performance validation. The images had an average resolution of approximately 3000 × 7000 pixels and were composed of 24-bit grayscale JPEGs. Before usage, all images were anonymized for privacy protection.

**Figure 1:**
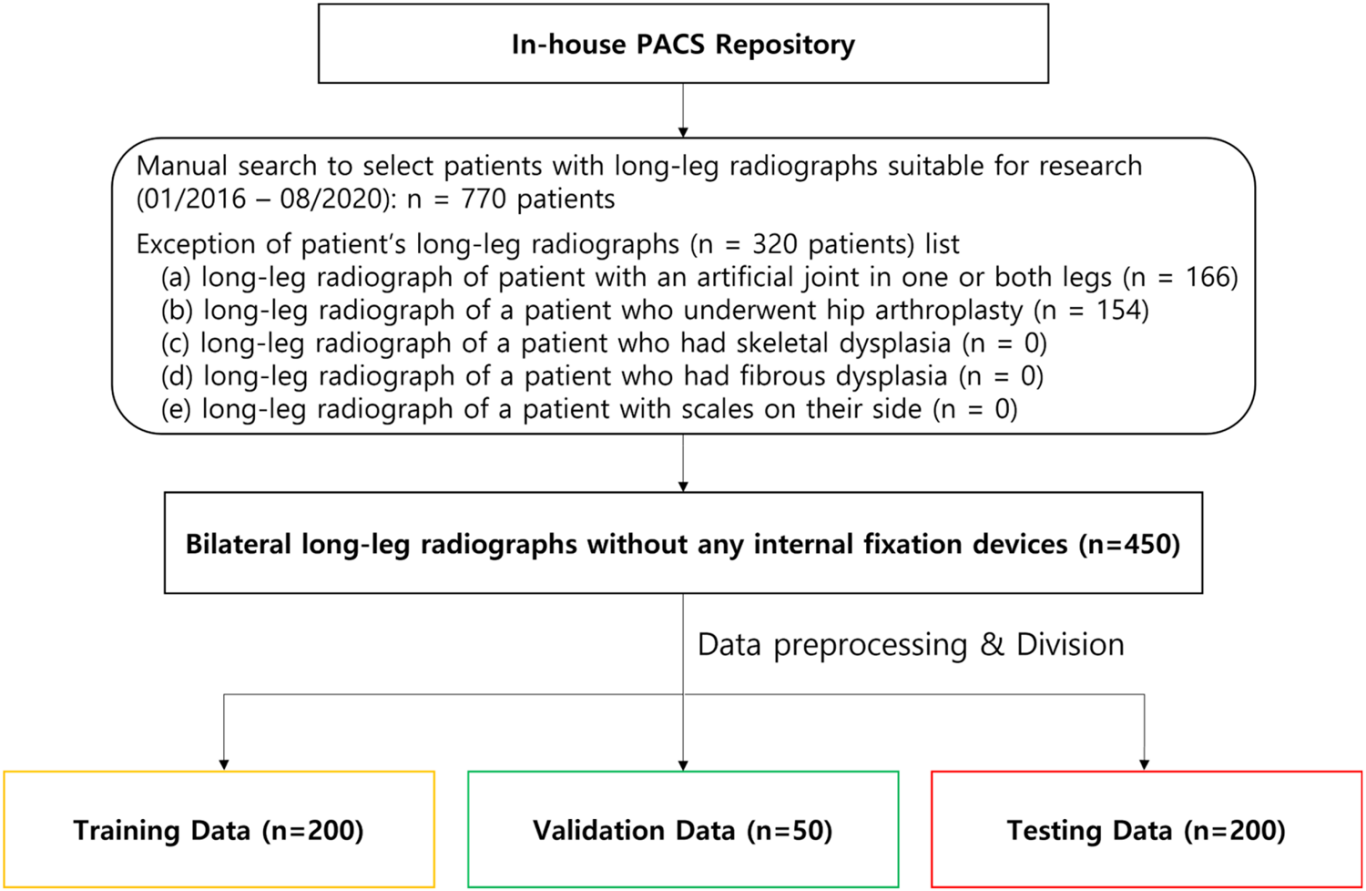
Flowchart detailing the number of patients included and excluded for this study based on given criteria.

The dataset was divided into training, validation, and test sets. For object detection and image segmentation training, data were allocated at the ratio training: validation: test = 200:50:200, with random selection for strict separation. The training set was used for object detection and semantic segmentation training to extract the required ROIs and mask images. The validation set was used to verify the performance of each model. The test set was used for the performance evaluation of the completed DL models and comparing the measurements of lower limb parameters obtained using the system with the clinical observations. In the ROIs within the individual radiographs, the segmentation masks were manually annotated by a board-certified radiologist (M.L., with 18 years of experience). These annotations were used as the ground truth for ROI detection and segmentation. Figure 1 displays the flowchart for the dataset composition process, and Table 1 summarizes the basic information of the patients from whom the dataset was acquired.

**Table 1:** Characteristics of the participating patients.

	Training set	Validation set	Test set
Total no. of patients	200	50	200
No. of male patients	73	20	75
No. of female patients	127	30	125
Mean age (year)	56.47	57.68	54.09
Age range (year)	12–88	21–77	11–89
Leg length (mm)	803.98 ± 60.32	805.95 ± 62.39	836.66 ± 61.94
Femoral length (mm)	457.76 ± 76.36	457.39 ± 45.89	474.34 ± 56.33
Tibial length (mm)	363.79 ± 29.61	363.58 ± 38.90	363.35 ± 29.65

## Reference standard for lower limb alignment

A board-certified radiologist (M.L., with 18 years of experience) measured parameters for assessing lower limb alignment in all radiographs within the test set. The measured parameters are listed in Table 2^20,21^.

**Table 2:** Parameters required to determine lower limb alignment status and normal range for each parameter.

Parameter	Normal range
Mechanical lateral proximal femoral angle (mLPFA)	85°–95°
Mechanical lateral distal femoral angle (mLDFA)	85°–90°
Mechanical medial proximal tibial angle (mMPTA)	85°–90°
Mechanical lateral distal tibial angle (mLDTA)	86°–92°
Mechanical axis deviation (MAD)	0–3 mm
Joint line convergence angle (mJLCA)	0°–2°
Mechanical tibiofemoral angle (mTFA)	0°–3°
Anatomical medial proximal femoral angle (aMPFA)	80°–89°
Anatomic lateral distal femoral angle (aLDFA)	79°–83°
Neck shaft angle (NSA)	124°–136°
Anatomical medial proximal tibial angle (aMPTA)	85°–90°
Anatomic lateral distal femoral angle (aLDFA)	79°–83°
Anatomical tibiofemoral angle (aTFA)	0°–3°
Full leg length	–
Femoral length	–
Tibial length	–

### Model architecture

**Figure 2:**
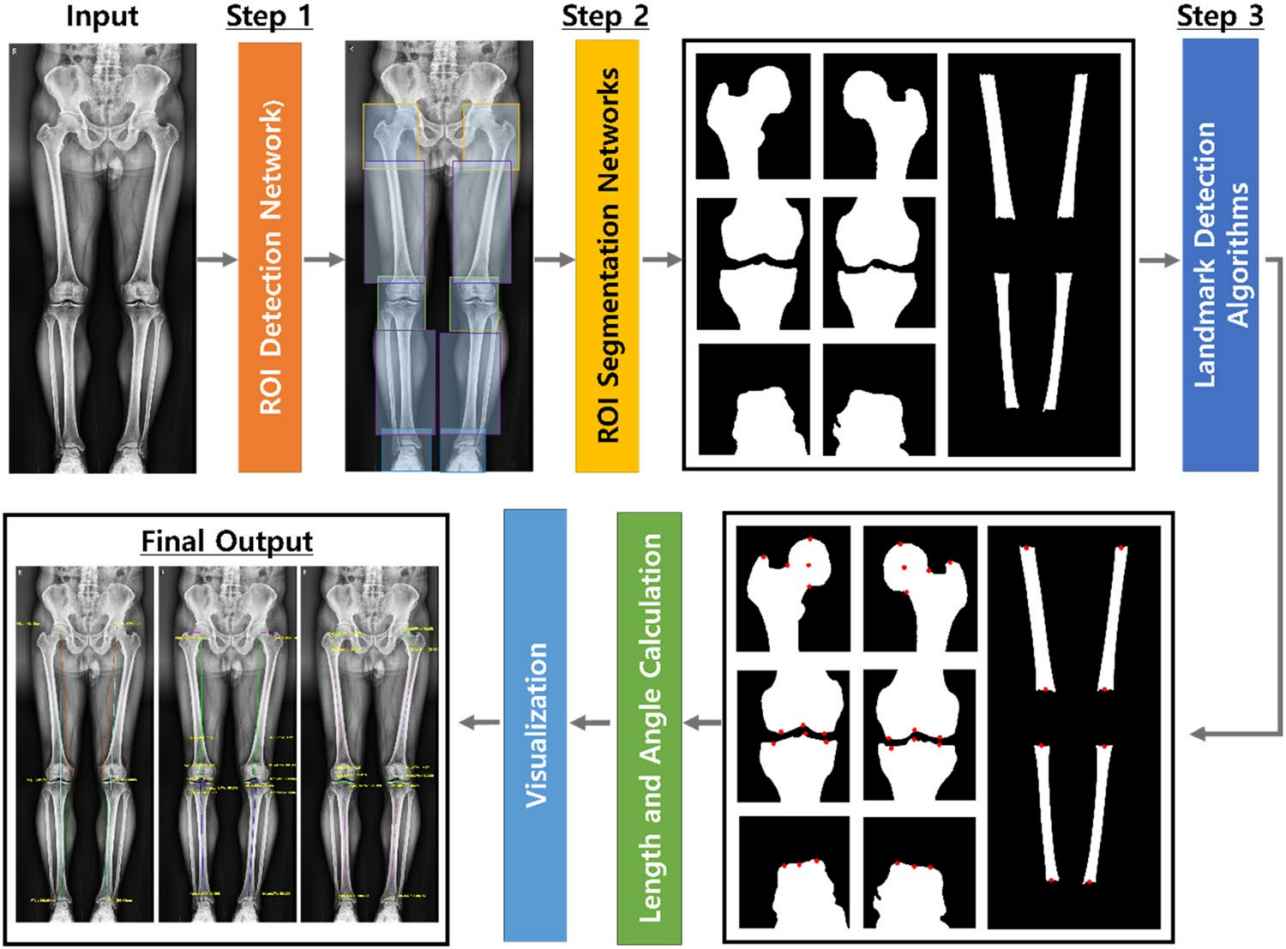
Progression of the proposed system.

The proposed measurement system consists of three steps, as summarized in Fig. 2. In step 1, a DL algorithm was used to detect the classes of ten ROIs corresponding to the left and right femurs and tibiae (four in total), femoral heads, knees, and ankles (six in total).

In step 2, the detected ROIs were used to extract mask images for the left and right femurs and tibiae long axes, femoral heads, knees, and ankles. Therefore, each ROI was cropped from the radiograph, and a semantic segmentation model was used to extract mask images.

In step 3, the generated mask images were used to measure the parameters for determining the lower limb alignment status, as shown in the radiographs. Therefore, an algorithm to detect the necessary landmarks was applied, wherein image processing techniques were used to determine landmarks based on medical definitions (see Supplementary Methods for details). The detected landmarks were used to calculate angles and lengths, and the results are shown in radiographs.

### Training strategy

First, the YOLOv5^22^ model was used to identify the ROIs for each part. To ensure its adequate training, the number of epochs and learning rate were set to 300 and 0.001, respectively. The Adam optimizer function, which is a common gradient-based optimization method, was used to perform weight updates using gradient descent. For training and inference, owing to a limited amount of graphics processing unit (GPU) memory, the input lower limb radiographs were resized to a fixed resolution of 640 × 480 pixels. Particularly, to eliminate image distortion from resizing, the input radiographs were padded to a square shape before being fed into the DL system. Finally, pixel values were normalized between 0 and 1. For the training dataset, the batch size was set to 8 and model validation was performed using the validation dataset at the end of each epoch to prevent overfitting.

Thereafter, the HarDNet-MSEG^23^ image segmentation model was used to create mask images for each ROI. Two-hundred epochs were used for training, with a learning rate of 0.005 and batch size of 1. Furthermore, the Adam optimizer function was used to perform weight updates using gradient descent. To compensate for the varying contrast levels in radiographs, contrast limited adaptive histogram equalization (CLAHE)^24^ was applied to each ROI to preserve local features while enhancing the low contrast of images. This method effectively distinguished important features and noise during the learning process. The CLAHE performance was influenced by two parameters, i.e., the block size for block-wise processing and clipping to prevent extreme pixel intensity variations within blocks^25^. Based on the best-performing experimental value, the block size and clipping value were set to (8,8) and 2.0, respectively. Because the performance improvement in ROI detection was negligible, CLAHE was not applied to full-leg radiographic images. The right long axis and ROIs for the femoral head, knee, and ankle were horizontally flipped to reflect the left direction data. Similar to dataset preparation for ROI detection, the extracted ROI images were padded to a square shape and resized to 512 × 512 pixels for training the image segmentation model. The model validation was performed using the validation dataset at the end of each epoch. An Intel(R) Xeon(R) Silver 4216 CPU @ 2.10 GHz and NVIDIA RTX 2080Ti were used for detection and image segmentation training, model performance evaluation, and inference time measurement.

### Statistical analysis

To evaluate the performance of DL model for ROI detection, the mean average precision (AP), which is a widely adopted evaluation metric for object detection, was used with intersection over union thresholds spanning from 0.5 to 0.95^26^. For bone segmentation, the Dice similarity coefficient (DSC)^27^ and Hausdorff distance (HD)^28^ were used as evaluation metrics. DSC measures the pixel-wise agreement between a predicted segmentation and its ground truth, and HD quantifies the largest discrepancy between two segmentation masks.

The reliability and accuracy of the proposed system were evaluated by calculating the concordance correlation coefficient (CCC), Pearson correlation coefficient (PCC), and intraclass correlation coefficient (ICC). Bland–Altman plots were used to investigate the similarity between the clinical and system measurements and the presence of biases.

Thereafter, the mean absolute deviation (MAD) was calculated to determine the variability and extent of differences between measurements performed by the radiologist and system. Finally, the mean absolute error (MAE), mean square error (MSE), and root mean square error (RMSE) were sequentially calculated to validate the measurement performance of the system.

## Results

### Study participants

The lower limb frontal radiographs obtained from 450 patients (mean age ± standard deviation, 55 ± 16; age range, 11–89 years; 168 men, 282 women) were used. The proportion of female patients was approximately twice of that of male patients, and the collected radiographs included those of the entire area from the hip to lower left and right ankles. However, images containing medical implants, skeletal anomalies, or bone dysplasia were excluded.

### Performance of DL models for ROI detection and segmentation

After training, the performances of the object detection and image segmentation models were tested using a predefined test dataset. According to the experimental results, detection failures were not observed for any test data, and the mean AP across ROIs was approximately 0.99, indicating a considerably high detection performance. Thereafter, DSC and HD were measured to evaluate the performance of the image segmentation model (HarDNet-MSEG) for femoral heads, knees, ankles, and shafts for femur and tibia. The average DSC for each class was observed to be 0.97. Table 3 summarizes the performance results of the object detection and image segmentation models.

**Table 3:** Performance of DL models for ROI detection and segmentation.

	ROI Class	AP	DSC	HD (mm)
Left side	Femoral shaft	1.00	0.97	5.93 ± 56
	Tibial shaft	1.00	0.97	4.12 ± 3.05
	Femoral head	0.99	0.97	6.16 ± 2.13
	Knee joint	0.99	0.97	6.91 ± 3.03
	Ankle	0.99	0.98	5.90 ± 2.23
Right side	Femoral shaft	1.00	0.97	5.83 ± 2.85
	Tibial shaft	1.00	0.97	4.73 ± 3.56
	Femoral head	0.98	0.98	7.21 ± 3.88
	Knee joint	0.99	0.97	7.37 ± 3.72
	Ankle	0.99	0.97	6.58 ± 3.76
Mean of the 10 ROIs’ average precision		0.99	–	–
Mean of the 10 ROIs’ DSCs		–	0.97	–

### Accuracy and reliability of parameter measurements

The 13 quantitative parameters for diagnosing lower limb malalignments, including the lengths of the two legs, femurs, and tibiae, revealed high concordance and close correlations with the measurements performed by the radiologist. For lower limb length measurements, the ICC, PCC, and CCC indicated high values for the entire lower limb length (0.979, 0.996, and 0.979), tibial length (0.905, 0.975, and 0.906), and femoral length (0.979, 0.986, and 0.940). Similarly, for the lower limb alignment assessment parameters, the experimental results indicated that the proposed system produced significantly reliable results with correlation coefficients greater than or equal to 0.9. As summarized in Table 4, the high measurement accuracy of the proposed system was observed using MAE, MSE, and RMSE, which validated its effectiveness.

**Table 4:** Correlation coefficients and error indices between the actual values observed by the radiologist and those measured by the system.

Parameter	Concordance correlation coefficient	Pearson correlation coefficient	Intraclass correlation coefficient	Mean absolute error	Mean square error	Root mean square error
mLPFA (°)	0.984	0.988 (< .001)	0.985	0.76	0.85	0.92
mLDFA (°)	0.969	0.970 (< .001)	0.969	0.53	0.40	0.63
mMPTA(°)	0.975	0.978 (< .001)	0.976	0.63	0.75	0.87
mLDTA (°)	0.910	0.925 (< .001)	0.920	0.87	2.14	1.46
MAD (mm)	0.991	0.991 (< .001)	0.991	0.80	1.69	1.30
mJLCA (mm)	0.943	0.944 (< .001)	0.944	0.51	0.40	0.63
mTFA (°)	0.980	0.980 (< .001)	0.980	0.46	0.29	0.54
aMPFA (°)	0.995	0.995 (< .001)	0.995	0.46	0.30	0.55
aLDFA (°)	0.995	0.977 (< .001)	0.978	0.46	0.29	0.54
NSA (°)	0.993	0.993 (< .001)	0.993	0.49	0.32	0.57
aMPTA (°)	0.981	0.981 (< .001)	0.981	0.56	0.62	0.79
aLDTA (°)	0.990	0.990 (< .001)	0.991	0.50	0.33	0.57
aTFA (°)	0.977	0.978 (< .001)	0.978	0.49	0.33	0.57
Full leg-length (mm)	0.979	0.996 (< .001)	0.979	1.20	1.16	1.08
Femoral length (mm)	0.940	0.986 (< .001)	0.979	1.71	1.74	1.32
Tibial length (mm)	0.906	0.975 (< .001)	0.905	1.33	1.33	1.15

**Figure 3:**
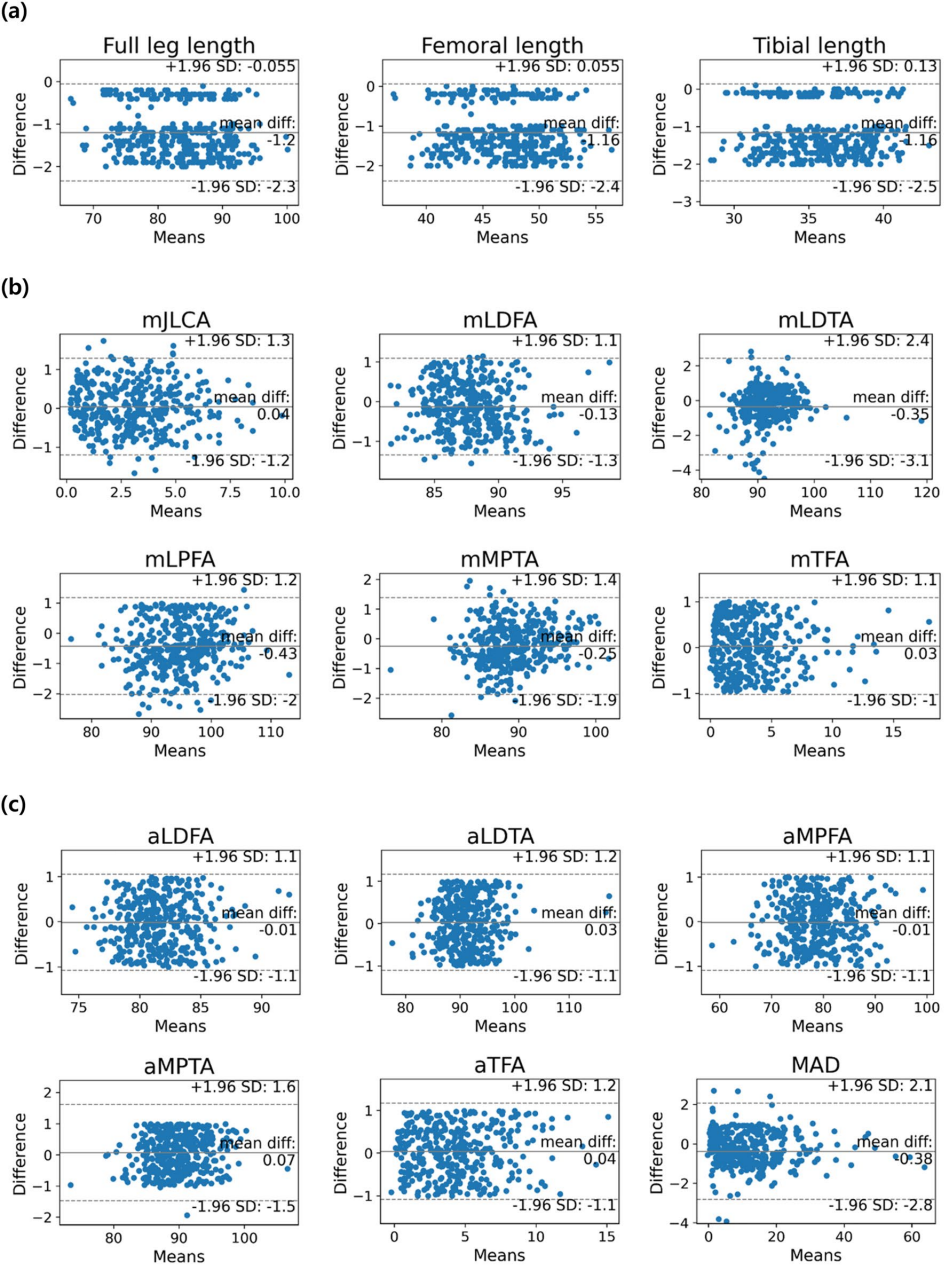
Bland–Altman plot for the reference standards and measurements using the DL-based system for each parameter. The x-axis represents the mean of the reference and corresponding parameter measured by the system, whereas the y-axis represents the difference between the two measurements. (**a**) Lower limb length, (**b**) parameters based on mechanical axes, and (**c**) parameters based on anatomical axes.

The Bland–Altman plots demonstrated that the measurements obtained using the system excellently agreed with the reference standard obtained by radiologist (Fig. 3). Particularly, marginal variability and mean difference were observed between the two measurements. Furthermore, a few observations deviated from the central line in certain parameters; however, these deviations were negligible.

**Figure 4:**
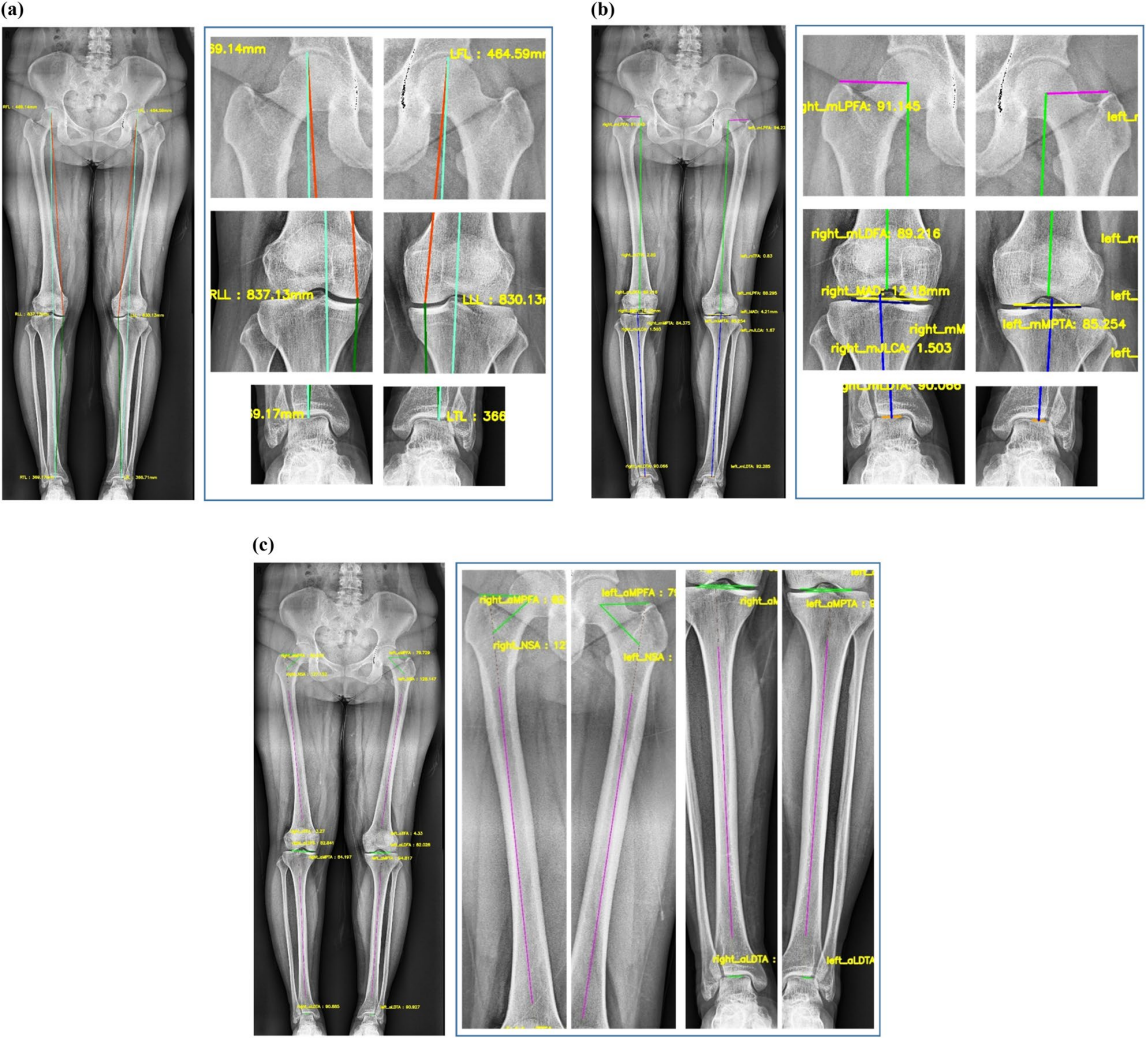
Example outputs of the system. The lines were generated using the DL-based automatic measurement system. Images on the right side indicate that the system accurately and reliably localized relevant landmarks required to assess lower extremity alignment. (**a**) Lower limb length, (**b**) parameters based on mechanical axes, and (**c**) parameters based on anatomical axes.

Consequently, MAD was analyzed by calculating the average of deviations obtained after subtracting the overall mean of the measurements from individual measurements to determine the variability using reference values. Table 5 summarizes these results, and Fig. 4 shows the cases of lower extremity alignment calculated using the DL-based system. Overall, these experimental results indicate the absence of systematic bias between the reference standard and parameters measured using the proposed system.

**Table 5:** Mean, mean difference, and mean absolute deviation for the actual values observed by the radiologist and the values measured by the system.

Parameter	Mean (standard deviation, SD)		Ours vs. Radiologist	
	Ours	Radiologist	Mean difference ± SD (95% CI)	MAD (95% CI)
mLPFA (°)	94.5 (± 5.30)	94.9 ( ± 5.22)	− 0.43 ± 0.81 (− 2, 1.2)	0.39 (0.34, 0.45)
mLDFA (°)	87.6 (± 2.53)	87.8 (± 2.57)	0.13 ± 0.62 (− 1.3, 1.1)	0.29 (0.25, 0.32)
mMPTA (°)	89.0 (± 4.00)	89.3 (± 3.87)	− 0.25 ± 0.83 (− 1.9, 1.4)	0.38 (0.3, 0.43)
mLDTA (°)	91.3 (± 3.73)	91.7 (± 3.57)	− 0.35 ± 1.42 (− 3.1, 2.4)	0.64 (0.52, 0.75)
MAD (mm)	12.0 (± 9.85)	12.3 (± 9.85)	− 0.38 ± 1.24 (− 2.8, 2.1)	0.47 (0.37, 0.57)
mJLCA (mm)	3.1 (± 1.88)	3.0 (± 1.91)	0.04 ± 0.63 (− 1.2, 1.3)	0.29 (0.25, 0.33)
mTFA (°)	3.3 (± 2.69)	3.2 (± 2.70)	0.03 ± 0.53 (− 1,1.1)	0.24 (0.21, 0.26)
aMPFA (°)	78.8 (± 5.66)	78.8 (± 5.64)	− 0.01 ± 0.55 (− 1.1, 1.1)	0.24 (0.22, 0.27)
aLDFA (°)	81.6 (± 2.57)	81.6 (± 2.55)	− 0.01 ± 0.54 (− 1.1, 1.1)	0.24 (0.22, 0.27)
NSA (°)	128.3 (± 4.96)	128.3 (± 4.90)	− 0.01 ± 0.57 (− 1.1, 1.1)	0.24 (0.22, 0.27)
aMPTA (°)	89.8(± 4.10)	89.7(± 4.03)	0.07 ± 0.78 (− 1.5, − 1.6)	0.29 (0.24, 0.35)
aLDTA (°)	91.2 (± 4.30)	91.2 (± 4.27)	0.03 ± 0.57 (− 1.1, − 1.2)	0.24 (0.22, 0.27)
aTFA (°)	4.2 (± 2.75)	4.2 (± 2.74)	0.04 ± 0.57 (− 1.1, 1.2)	0.25 (0.22, 0.28)
Full leg-length (mm)	834.5 (± 62.17)	836.1 (± 63.76)	− 1.2 ± 0.58 (− 2.3, − 0.055)	0.47 (0.42, 0.61)
Femoral length (mm)	471.3 (± 30.31)	474.2 (± 37.27)	− 1.16 ± 0.62 (− 2.4, − 0.055)	0.50 (0.44, 0.65)
Tibial length (mm)	361.3 (± 29.55)	362.8 (± 29.36)	− 1.16 ± 0.66 (− 2.5, 0.13)	0.54 (0.48, 0.70)

Finally, the duration at each step of the system and that required to compute the final outputs were measured (Table 6). Measurements were repeatedly performed for a single lower limb radiograph, and separate measurements were performed for central processing unit (CPU)-only and combined GPU–CPU computations. For CPU-only computations, the average durations for the first, second, and third steps were approximately 1.08, 8.59 and 2.53 s, respectively. For combined CPU-GPU computations, the average durations for the first, second, and third steps were 3.43, 7.95, and 2.7 s, respectively. For CPU-only and combined CPU-GPU computations, the total execution times for the system were approximately 12.2 and 14.08 s, respectively, and the variation in these two values can be attributed to the time required to load DL models into the GPU memory. Particularly, while measuring the execution time of the system after loading the DL models into GPU memory for ROI detection and segmentation, it was observed that steps 1 and 2 preserved time. Consequently, compared to CPU-only computations, no significant difference was observed in the overall execution time.

**Table 6:** Comparison of average and total measurement times for each step of the proposed system obtained with and without using GPU.

	1st Step	2nd Step	3rd Step	Total
Without GPU	1.08 s	8.59 s	2.53 s	12.20 s
With GPU	3.43 s	7.97 s	2.52 s	13.92 s
With GPU*	2.21 s	7.66 s	2.51 s	12.38 s

*Indicates the case where deep-learning models have been already loaded into the GPU memory.

## Discussion

This study proposed a method for automated measurements of bilateral leg lengths, femoral and tibial lengths, and parameters used to determine the presence of lower limb malalignments by applying DL technology to anteroposterior lower limb radiographs. First, the ability of the DL-based model to detect and segment the femur, tibia, femoral head, knee, and ankle was validated for measuring each parameter using a predefined test dataset of 200 images (average of class-wise AP = 0.99; average DSC = 0.97), indicating excellent performance.

Comparing the results of each system-calculated indicator with the values observed by a radiologist revealed that the measurements of the total lower limb length, femoral and tibial lengths, and the 13 parameters for determining lower limb alignment (mLPFA, mLDFA, mMPTA, mLDTA, MAD, mJLCA, mTFA, aMPFA, aLDFA, NSA, aMPTA, aLDTA, and aTFA) exhibited a significantly high correlation. (CCC, ICC, and PCC $\ge$ 0.91) To our knowledge, this study is the first to evaluate such a large number of alignment indicators. Furthermore, the obtained MAE, MSE, and RMSE results revealed the absence of significant bias between the observed values and observations of an actual evaluator.

Schock et al.^12^ proposed an automated evaluation method for the lower limb alignment status by applying DL technology to anteroposterior lower limb radiographs, thereby measuring the alignment indicators rapidly and accurately (HKAA: PCC = 0.99 [*P*-value < 0.001], ICC = 0.99, and mean ± deviation = 0.10 ± 4.42; AMA: PCC = 0.99 [*P*-value < 0.001], ICC = 0.89, and mean ± deviation = 5.13 ± 1.36). Similar to the proposed method, an image segmentation model was used to generate mask images for the entire femur and tibia, thereby directly identifying points required to calculate the indicators using the contours of these mask images. However, this method is considerably different from the proposed method wherein the precise landmark locations are determined using the entire mask images of the femur and tibia extracted using the image segmentation model. To achieve this, the ROIs in the radiographs are identified, the corresponding mask images are extracted, and the landmarks within those regions are determined. This approach considers local features that can be easily overlooked in the entire image. Therefore, these factors contribute to the performance variations between systems.

Comparing the results obtained using the proposed method with those observed by the radiologist revealed that the proposed method has high concordance and reliability. Furthermore, the time required to automatically generate results using a single radiograph input was approximately 12 s, which was faster than that required for radiologists to perform direct observations (approximately 130 s per image). Therefore, a large number of images can be measured accurately at a substantially faster pace, which enables the swift determination of the lower limb alignment status and renders the model effective for repetitive measurement tasks required for prognosis observation.

However, this methodology has several limitations. First, the evaluation process after obtaining the alignment indicators was performed using a limited internal dataset. Second, the data did not include radiographs with abnormal skeletal structures or bone dysplasia. To handle diverse patient groups, the development of sophisticated DL models and establishment of large datasets including these patients, are necessary. Third, a single evaluator obtained the validation data; however, methods such as comparing the system to the results of the same evaluator measuring the same radiographs with a time interval or introducing one or more additional evaluators for cross-validation can be considered.

In summary, a DL-based system was developed and evaluated for measuring various parameters to assess lower limb malalignments, including angle and length. Because the steps involved in the measurement process are executed automatically without human intervention, the process can be performed rapidly. This is beneficial for reducing the workload of clinicians and establishing appropriate treatment plans for patients through fast and accurate diagnoses, thereby enhancing the effectiveness of treatments. Further prospective studies should be performed to extend this system by increasing the diversity of measured parameters and patient groups, including those with artificial joints and skeletal abnormalities.

### Supplementary Information

Supplementary Information.

## Data Availability

The datasets generated and/or analyzed during the current study are not publicly available owing to specific institutional requirements governing privacy protection. However, they may be available from the corresponding author on reasonable request.
