# Supplementary material for: A deep learning approach for fully automated measurements of lower extremity alignment in radiographic images
Source: Sci Rep. 2023 Sep 6;13:14692. doi: 10.1038/s41598-023-41380-2 (PMC10482837; doi:10.1038/s41598-023-41380-2)
Supplement: Supplementary file 1 — Supplementary Information. [file 41598_2023_41380_MOESM1_ESM.docx]

Supplementary Material

**S1. Supplementary Methods**

This section presents the detailed description of landmark detection algorithms. For brevity, it is assumed that the algorithms localize relevant landmarks on the left leg.

**Landmark detection in the femoral region**

The landmarks extracted from the femoral region consist of a total of 5 pixels corresponding to the top and center of the femoral head, two points on the femoral neck, and top of the greater trochanter. The contour of the femoral region is extracted using its binary mask obtained from step 2. Assuming that the x-y coordinate of the top-most left pixel is (0,0), the pixel with the smallest y-coordinate is selected as the top of the femoral head. For several pixels with the same y-coordinate, the medial pixel is selected as the landmark. Thereafter, the two landmarks corresponding to the femoral neck are extracted using the convex hull algorithm [1] . First, the algorithm extracts convex regions from the femoral head contour and determines convex defects. Second, the farthest two points in the set of convex defects are selected and designated as the femoral neck. To determine the center of the femoral head, a line connecting the two pixels corresponding to the femoral neck (e.g., *y = ax + b*) is drawn, and the pixel corresponding to the center of area composed of pixels within the mask that satisfy the condition (e.g., *y > ax + b*) is selected as the landmark. Finally, among the two pixels corresponding to the femoral neck, the pixel with the smaller y-coordinate is selected and the binary mask contour is traversed in the direction of increasing x-coordinate starting from the pixel. Using the traversed pixels, the pixel with the greatest y-coordinate is selected as the top of the greater trochanter.

**Landmark detection in the knee joint**

The knee joints contain six relevant landmarks, including intercondylar notch, lateral and medial condyles of the femur, intercondylar eminence, lateral and medial condyles of the tibia. Among the coordinates of distal femur contour, the x-coordinate of intercondylar notch is computed as that of the midpoint between the two pixels corresponding to the maximum and minimum x-coordinates, respectively. Thereafter, based on the selected x-coordinate, the pixel with the greatest y-coordinate on the contour is selected as the intercondylar notch. Among the pixels located to the left and right contour of intercondylar notch, the pixels corresponding to the greatest y-coordinate are selected as the medial and lateral condyle of the femur, respectively. The landmark detection methods for the tibia and femur are approximately identical. However, they differ in the detection of the y-coordinate of landmarks because each bone exists in the proximal and distal regions.

**Landmark detection in the femoral and tibial shafts**

The proposed system requires four landmarks for femoral and tibial shafts. The smallest bounding boxes enclosing the binary masks for individual shafts are first identified. The center points of the shorter side of the rectangles are computed and set as the required landmarks.

**Landmark detection in the ankle joint**

For the ankle joint, the center, medial, and lateral aspects of the talar dome are required. To locate these landmarks, the most convex points on the left and right sides of the talus need to be identified. This is achieved by determining the minimum polygon enclosing the talus. Among the vertices of the obtained polygon and using the center point of the mask, the pixels with the smallest y-coordinates on the left and right sides are selected. After joining the two identified convex points by a line, the y-coordinates of the pixels at the ¼th and ¾th points are decreased by unity. Therefore, the points of intersection with the ankle outline are identified, which are selected as the medial and lateral aspects of talar dome. Furthermore, the line connecting the two convex points is used to find the center coordinate of talar dome, and the y-coordinate is decreased by unity to designate the point of first intersection with the ankle contour as the landmark.

**References**

1. M.A. Jayaram and Hasan Fleyeh. (2016). Convex Hulls in Image Processing: A Scoping Review. American Journal of Intelligent System, 6(2), pp. 48-58.
